# A20 functions as mediator in TNFα-induced injury of human umbilical vein endothelial cells through TAK1-dependent MAPK/eNOS pathway

**DOI:** 10.18632/oncotarget.18191

**Published:** 2017-05-25

**Authors:** Lei Li, Bingqing Huang, Shiyang Song, Hareshwaree Sohun, Zhiheng Rao, Luyuan Tao, Qike Jin, Jingjing Zeng, Rongzhou Wu, Kangting Ji, Jiafeng Lin, Lianpin Wu, Maoping Chu

**Affiliations:** ^1^ Institute of Cardiovascular Development and Translational Medicine, The Second Affiliated Hospital & Yuying Children’s Hospital, Wenzhou Medical University, Wenzhou 325027, China; ^2^ Shanghai Xinrui Medical Center, Shanghai 200020, China

**Keywords:** TNFα, TAK1, eNOS, A20, HUVECs

## Abstract

A20, a negative regulator of nuclear factor κB signaling, has been shown to attenuate atherosclerotic events. Transforming growth factor beta-activated kinase 1 (TAK1) plays a critical role in TNFα-induced atherosclerosis via endothelial nitric oxide (NO) synthase (eNOS) uncoupling and NO reduction. In the study, we investigated the hypothesis that A20 protected endothelial cell injury induced by TNFα through modulating eNOS activity and TAK1 signalling. Human umbilical vein endothelial cells (HUVECs) were stimulated by TNFα. The impact of A20 on cell apoptosis, eNOS expression and NO production and related TAK1 pathway were detected. Both eNOS and NO production were remarkably reduced. TAK1, p38 MAPK phosphorylation and HUVECs apoptosis were enhanced after TNFα stimulation for 2 hrs. Inhibition of A20 significantly activated TAK1, p38 MAPK phosphorylation, and cell apoptosis, but blocked eNOS expression and NO production. Furthermore, p38 MAPK expression was suppressed by A20 over-expression, but re-enhanced by inhibiting A20 or activation of TAK1. Furtherly, TNFα-induced suppression of eNOS and NO production were largely prevented by silencing p38 MAPK. Collectively, our results suggested that A20-mediated TAK1 inactivation suppresses p38 MAPK and regulated MAPK/eNOS pathway, which contributes to endothelial cell survival and function preservation.

## INTRODUCTION

The major causes of vascular diseases are endothelial injury, which is closely related with the atherosclerosis [1]. Endothelium derived nitric oxide (NO), a vasodilator molecule produced by the intact endothelial layer of the vascular wall, has a key role in vascular biology, functioning as an important regulator of vascular tone, leukocyte adhesion to microvascular endothelium, and capillary leakage [2–4]. NO bioavailability is highly associated with endothelial NO synthase (eNOS), an enzyme that generates the vasoprotective molecule NO [5]. Exposure to oxidative stress and inflammation induces the endothelium vasodilatation and endothelial cell injury through blocking the generation of eNOS [6]. The improvement of blood gradient and decreased vascular injury were reported in loss of eNOS in mice [7–8].

TNF-α is an important inflammatory factor in early atherosclerosis [9]. A20, tumor necrosis factor–induced protein 3 is a negative regulator of nuclear factor κB signaling, could play the anti-atherosclerosis role by inhibiting the inflammatory factors [10–11]. A20 effectively suppressed inflammatory, vascular injury, cell apoptosis and atherosclerosis formation. Plenty of studies demonstrated that A20 also ameliorated endothelial injury through directly upregulating eNOS expression and NO generation [12–13], but the molecular mechanism was undetermined.

Transforming growth factor beta-activated kinase 1 (TAK1) has a crucial role in the atherosclerosis, and not only involved with maturity and shift of dendritic cell (DCs), but also regulated NO generation [14–15]. A20 could depress vascular injury through mediating p38 MAPK signalling [16–17]. Conversely, TNFα-induced atherosclerosis could also be enhanced by activating TAK1 [18]. TAK1 deletion improved oxidative stress, increased eNOS activation and NO production after TNFα stimulation [19]. However, there is no further investgation about the role of A20 in regulating TAK1 activity in endothelial injury induced by TNFα. Therefore, we speculated that A20 might ameliorate TNFα-induced endothelial impairment through regulating TAK1. In the present study, we deciphered the effect of A20 on TAK1/eNOS/NO pathway in endothelial function.

## RESULTS

### A20 mediated eNOS expression in HUVECs induced by TNFα

**Figure 1:**
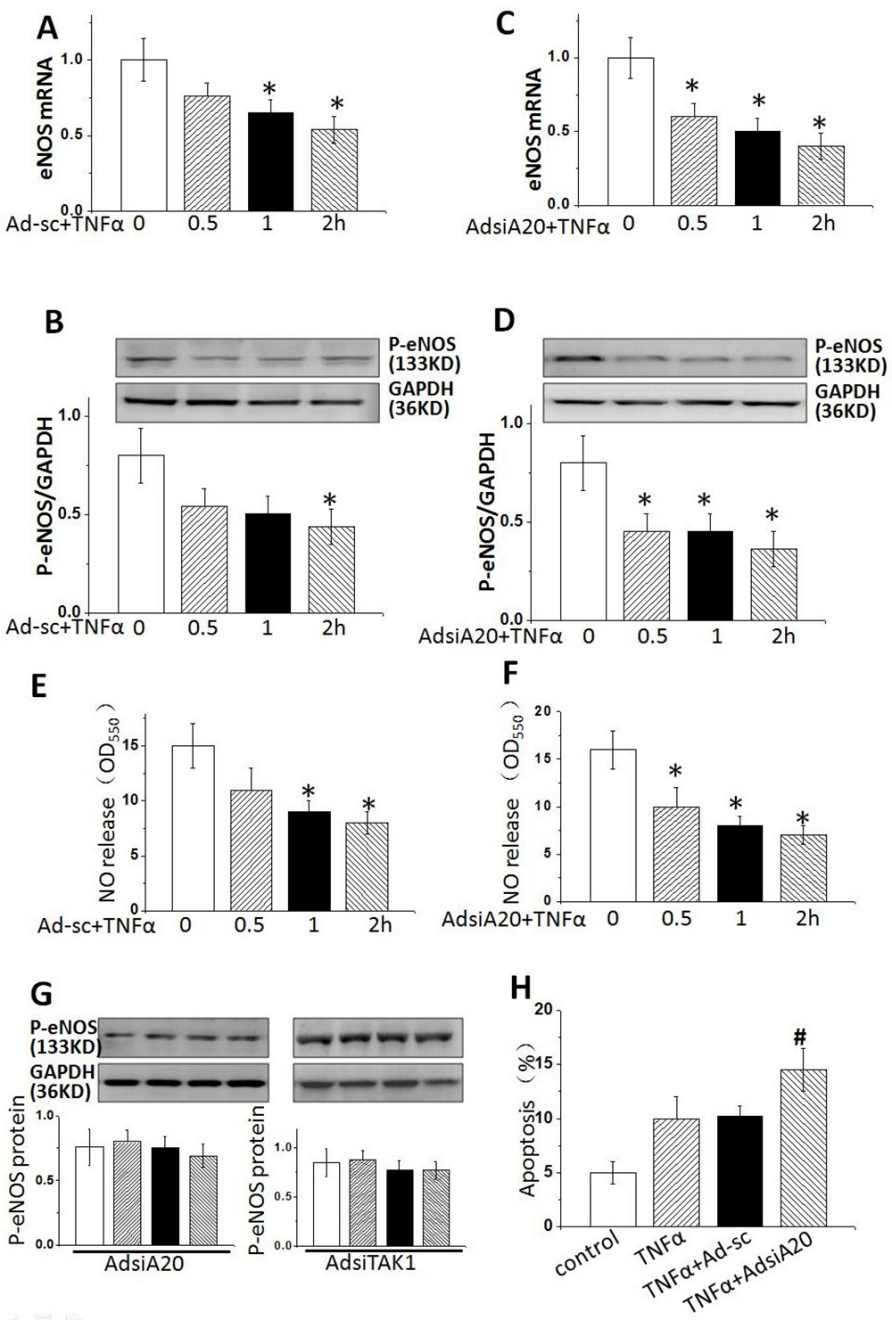
Knockdown A20 reduced eNOS expression in TNFα -stimulated HUVECs HUVECs were treated by TNFα (20 ng/ml) for 0, 30, 60 and 120 mins with or without AdsiA20. **(A** and **B)** The mRNA and protein levels of phosphate-eNOS (p-eNOS) were detected by real-time PCR and Western blotting. **(C** and **D)** p-eNOS expression with pre-treatment of AdsiA20 or TNFα. **(E** and **F)** Relative quantification of nitric oxide production using nitrate reductase method in HUVECs pretreated with or without AdsiA20. **(G)** p-eNOS expression with pre-treatment of AdsiA20 or AdsiTAK1 in the absence of TNFα. **(H)** The percentage of apoptotic cells detected by TUNEL staining. *P < 0.05 versus TNFα-induced group at 0 h, respectively. Ad-sc: adenovirus-scramble siRNA;^#^*P*<0.05 versus TNFα-induced adenovirus-scramble siRNA group treated with 2 hours (n=6 independent experiments).

We first examined the function of A20 in TNFα -induced endothelial damage. HUVECs were treated with TNFα (20 ng/ml) for 2 hours in the presence or absence of pre-treatment with AdsiA20 or AdsiTAK1. Both mRNA and protein levels of phosphorylation-eNOS at site of ser1177 were detected by qPCR and WB. In a time-dependent way, TNFα markedly reduced eNOS expression (Figure 1A and 1B) and NO release (Figure 1E). Further, downregulation of eNOS, the transcriptional and post-transcriptional levels, was enhanced by AdsiA20 treatment for 36 hrs (Figure 1C and 1D), but AdsiA20 and AdsiTAK1 had no effect on the basal eNOS production in the absence of TNFα (Figure 1G). Considering that endothelial function was mostly dependent eNOS phosphorylation and NO production, we further investigated the effect of A20 on regulation of the activities of eNOS (Figure 1F). Our data showed that NO release induced by TNFα was time-dependently reduced, and A20-mediated NO production was largely dependent on the phosphorylation of eNOS.

Previous study showed that eNOS/NO pathway played a critical role in cell apoptosis [20–21], we therefore examined the effect of NO expression on HUVECs. Apoptosis was comparably lower in resting HUVECs, but the numbers of apoptotic cells were both remarkably increased after induction with TNFα for 2 hrs, and this effect could be enhanced by AdsiA20 pre-treatment (Figure 1H), indicating that A20-mediated eNOS pathway played a crucial role in improving HUVECs survival induced by TNFα.

**Figure 2:**
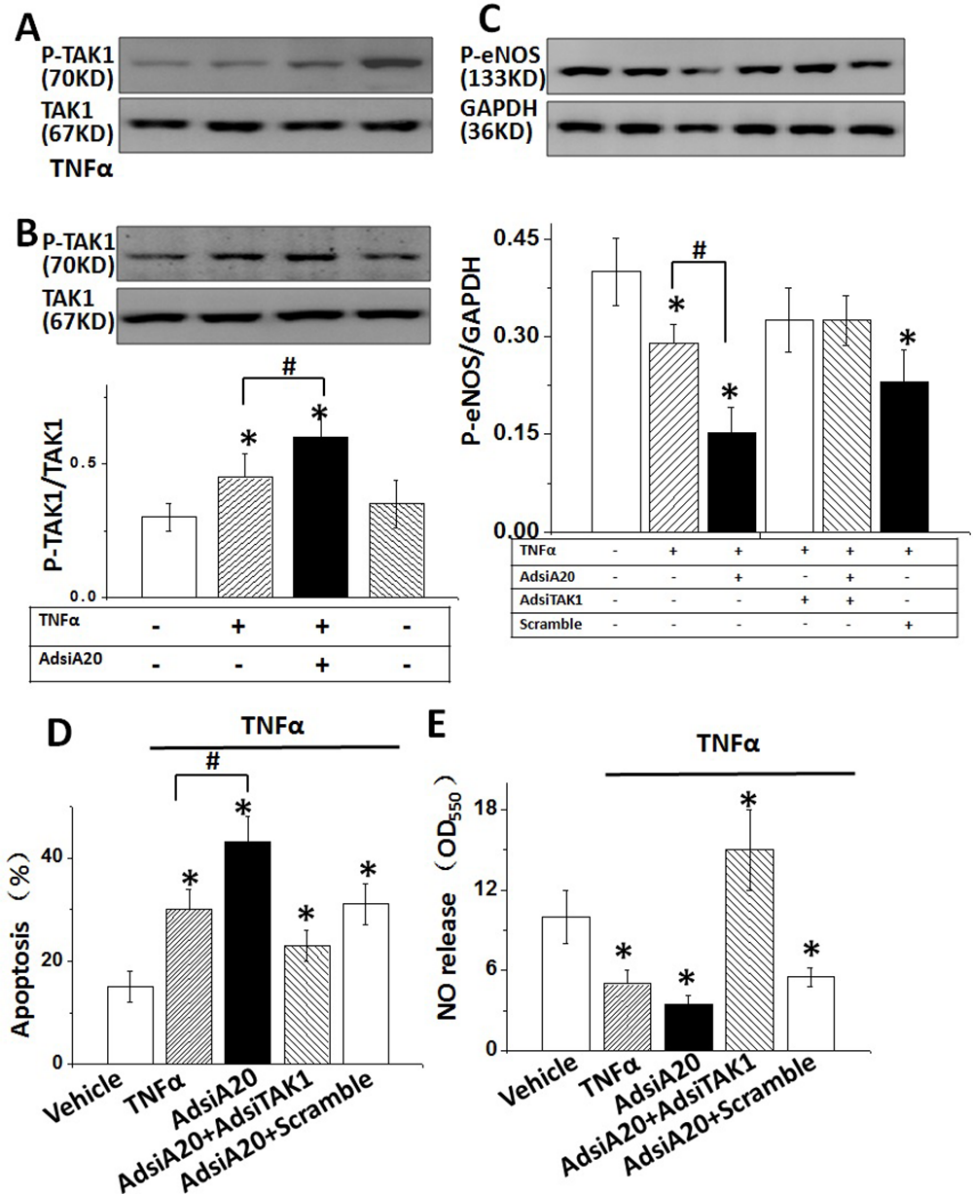
TAK1 inactivation was required for TNFα-induced p-eNOS expression **(A)** HUVECs were treated by TNFα (20 ng/ml) in a time-dependent manner, and the phosphorylation level of TAK1 was detected by Western blotting. **(B)** TAK1 phosphorylation in HUVECs after stimulation with TNFα for 2 hrs with or without AdsiA20 pre-treatment. **(C)** The effect of A20 on TNFα-induced p-eNOS expression with or without AdsiTAK1 (scramble siRNA as negative control). **(D)** The effect of A20 on TNFα-induced HUVECs apoptosis with or without AdsiTAK1 (scramble siRNA as negative control) measured by TUNEL staining. **(E)** Relative quantification of NO release using nitrate reductase method in HUVECs treated by different conditions. *P<0.05 versus control group, respectively (n=6 independent experiments).

Previous studies indicated an important role of TAK1 in regulating the expression of eNOS [15, 22]. Therefore, we investigated whether TAK1 was involved in TNFα-mediated suppression of eNOS, and whether TAK1 activity was affected by A20. TAK1 phosphorylation was examined in TNFα-stimulated HUVECs from 0 to 2hrs. The results showed that the level of phosphorylated TAK1 was greatly increased by TNFα stimulation at 2 hrs (Figure 2A). However, the boosting effect of TNFα on TAK1 activity was significantly enhanced by pre-treatment cells with AdsiA20 although AdsiA20 induced an increase of TAK1 phosphorylation in the absence of TNFα (Figure 2B), indicating a crucial role of A20 in reducing the baseline of TAK1 activity. Next, we knocked down TAK1 gene to investigate the impact of TAK1 silencing on eNOS expression. As expected, the eNOS expression was suppressed by TNFα, and re-enhanced by pre-treatment with AdsiTAK1 (Figure 2C). Furthermore, knockdown of TAK1 reduced apoptosis induced by TNFα (Figure 2D). We further detected this A20’s effect on NO synthesis in HUVECs. As expected, A20-mediated NO production in TNFα-induced HUVECs was increased by pre-treating cells with TAK1 siRNA (Figure 2E). These data indicated that TAK1 was critically involved in A20-mediated eNOS, NO production and against apoptosis.

### A20-mediated TAK1/eNOS pathway was A20 dependent

**Figure 3:**
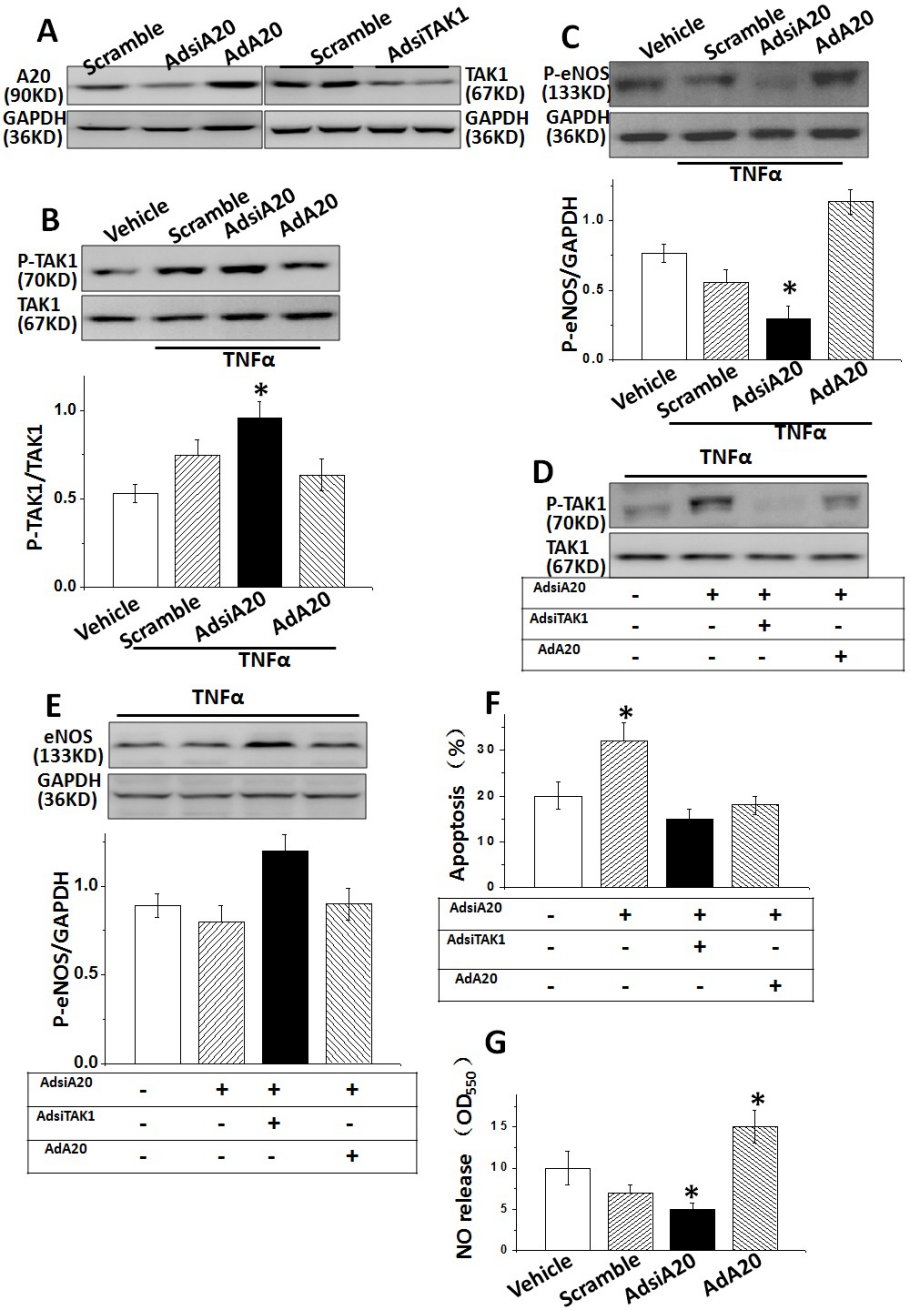
A20 was required for TNFα-caused TAK1/eNOS signalling in HUVECs **(A)** A20 or TAK1 expressions were examined in HUVECs pre-treated with AdsiA20 siRNA (it’s scramble siRNA as negative control), AdA20 or AdsiTAK1. **(B** and **C)** The expressions of TAK1 phosphorylation and p-eNOS were measured in HUVECs induced by TNFα, pre-treated by AdsiTAK1, or AdA20. **(D** and **E)** The effect of A20 on TNFα-induced TAK1 phosphorylation and p-eNOS expression after pre-treated with AdsiTAK1 or AdA20. **(F)** The effect of A20 on TNFα-induced HUVECs apoptosis after pre-treated with AdsiA20, AdA20 or AdsiTAK1. **(G)** Relative quantification of NO release using nitrate reductase method in HUVECs pre-treated by different conditions. *P<0.05 versus control Group, respectively (n=6 independent experiments).

We next investigated whether A20-mediated TAK1 activation and eNOS expression was A20 dependent. HUVECs were pre-treated with A20 siRNA and AdA20, respectively for 36 hrs, the expression of A20 or TAK1 was verified by WB (Figure 3A). A20 was significantly reduced in HUVECs by either siRNA transfection or increased after AdA20 incubation of the cell. Importantly, we found that both AdA20 pre-treatment could significantly reduced the TAK1 phosphorylation, inversely AdsiA20 led to a remarkable activation of TAK1 and reduction of eNOS in HUVECs (Figure 3B and 3C). Next, we examined the effect of A20 inhibition on A20-induced TAK1 activity and eNOS expression. Data showed that the inhibitory effect of A20 on TAK1 activation and eNOS expression was rescued by AdA20 treatment (Figure 3D and 3E). In addition, A20 siRNA treatment also suppressed the NO systhesis in HUVECs (Figure 3G). Although TNFα-induced cell apoptosis was reduced by AdA20 pre-incubation of the cell, this reduced apoptotic effect was attenuated by suppressing A20 with specific siRNA (Figure 3F).

### A20-required TAK1 inactivation suppressed p38 MAPK expression in HUVECs

**Figure 4:**
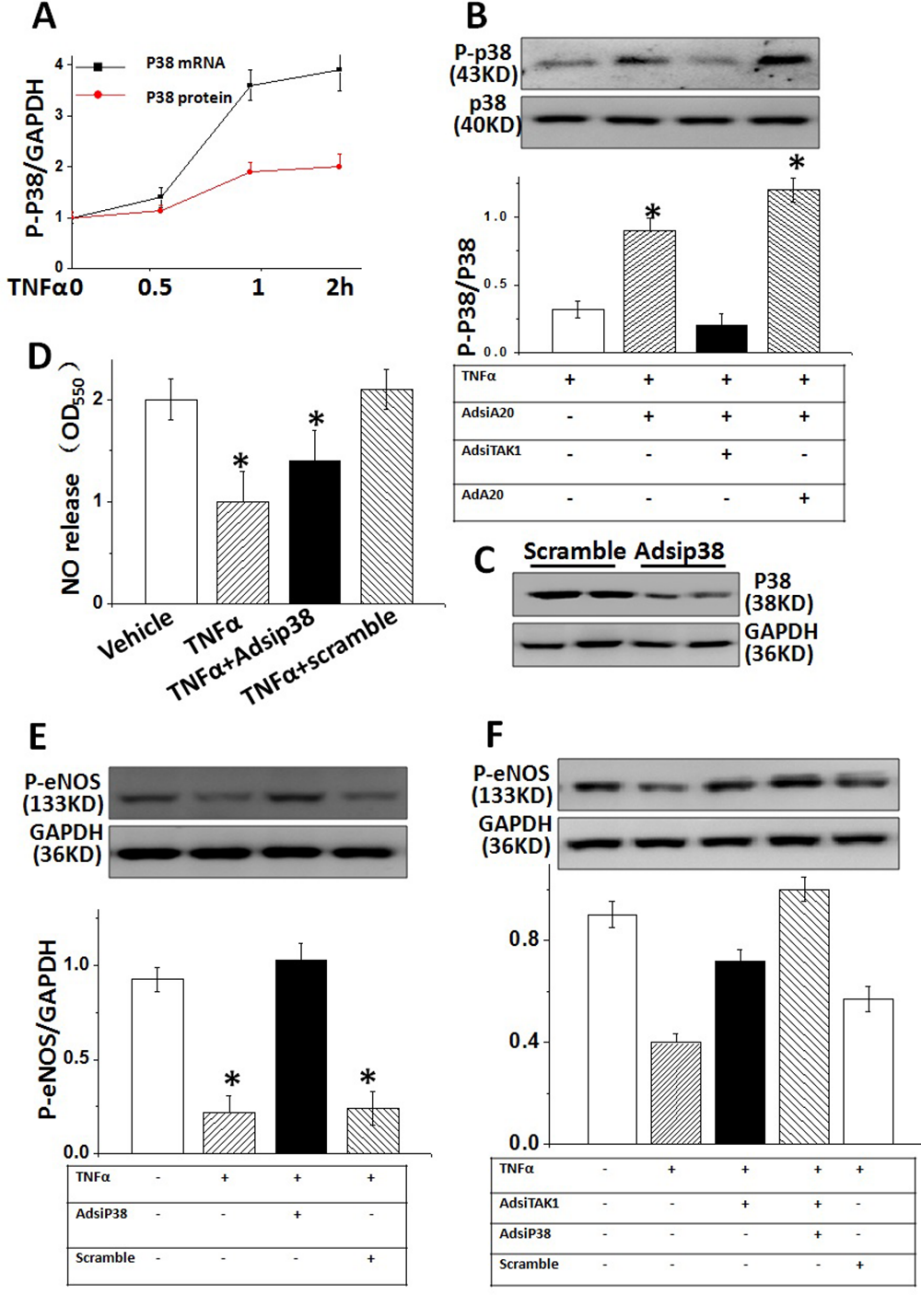
A20-dependent TAK1 activity reduced eNOS expression through regulating p38 MAPK in HUVECs **(A)** TNFα-caused phosphorylation p38 (p-p38) MAPK expression **(B)** The effect of A20 on TNFα-induced p38 MAPK protein levels pretreated with AdsiA20, AdsiTAK1 or AdA20, respectively. **(C)** P38 expressions were examined in HUVECs pre-treated with Adsip38 siRNA (it’s scramble siRNA as negative control) **(D)** The effect of Adsip38 MAPK on TNFα-induced p-eNOS expression in HUVECs. **(E)** Relative quantification of NO release in TNFα -treated HUVECs with or without pre-treatment of Adsip38 MAPK. **(F)** TAK1-mediated impact on eNOS occurs through the expression of p38 MAPK. Data represented typical results of 4 independent experiments.

**Figure 5:**
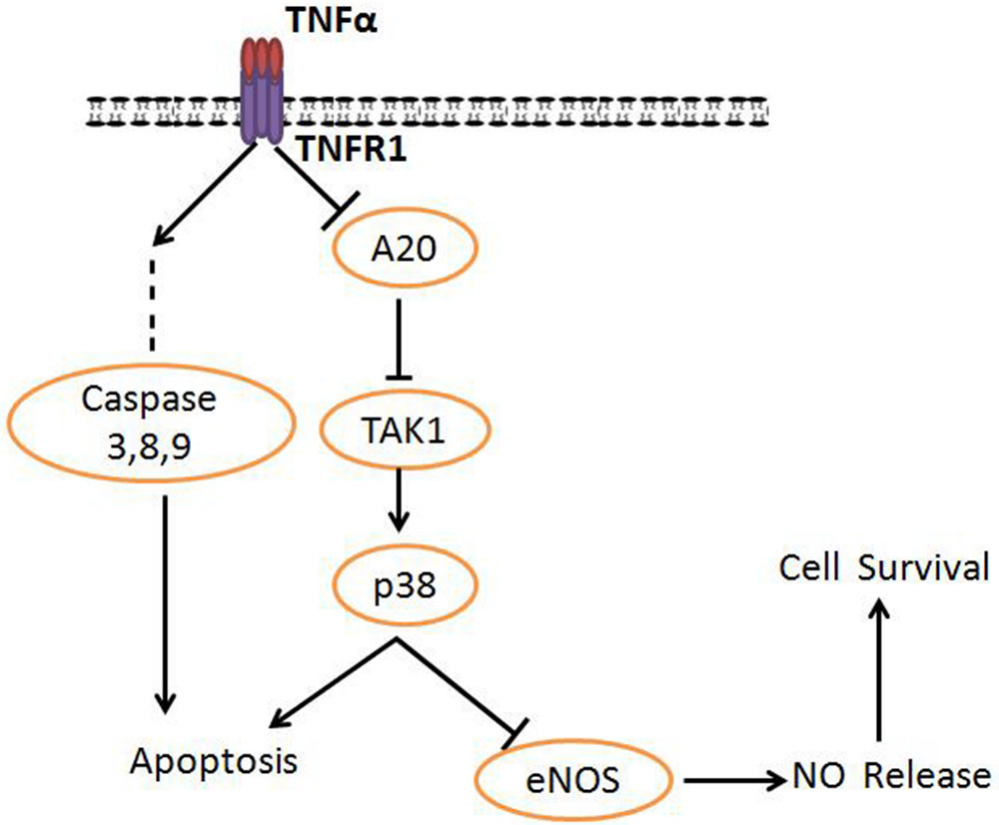
Proposed model A20 functions as a molecular mediator in tumor necrosis factor receptor-1 (TNFR1)–mediated cell survival/death signaling.

Activation of P38 MAPK had a crucial function in endothelial dysfunction [23]. To investigate whether A20 protected cultured HUVECs from TNFα injury through downregulating p38 MAPK expression *in vitro*, we detected the mRNA and protein expression of phosphorylation p38 (p-p38) MAPK in TNFα-stimulated HUVECs for 2 hrs. Both mRNA and protein levels of p-p38 MAPK were significantly enhanced after TNFα treatment, and expression of p-p38 MAPK was suppressed by AdA20 or AdsiTAK1 (Figure 4A and 4B), suggesting p-p38 MAPK expression was regulated through A20 control partially. Importantly, eNOS elevation was suppressed in TNFα-induced HUVECs, which were significantly reversed by inhibition of p38 MAPK with special siRNA against p-p38 MAPK (Figure 4C). Inhibition of p38 MAPK also significantly increased the NO release and eNOS phosphorylation (Figure 4E) in HUVECs which was suppressed by TNFα (Figure 4D) and enhanced by inhibition of TAK1(Figure 4E), suggesting that p-p38 MAPK was critical for TNFα-induced inhibition of eNOS activity, the upstream of NO production (Figure 5).

## DISCUSSION

TNFα played a critical role in the promotion of atherosclerosis by decreasing cellular cholesterol efflux from foam cells [15, 24]. Accumulated oxidative stress induced by TNFα led to the endothelial impairment and resulted in atherosclerotic lesions [25–26]. The previous studies showed a critical role of eNOS in the regulation of endothelial function and structure, and A20 could promote NO generating in endothelial cells [13, 27]. However, the involving mechanism for the molecular role of A20 in endothelium function was not well understood. In this study, we found that (1) A20 protected HUVECs from cell apoptosis induced by TNFα; (2) A20 promotes NO release through inhibition of TAK1 and enhancing eNOS expression; (3) TAK1 activation inhibited by A20 upregulates the p38 MAPK, and promotes NO synthesis. Taken together, our results suggest that A20 regulates TNFα-induced injury through TAK1-dependent AMPK/eNOS pathway in HUVECs (Figure 5).

TAK1 is a serine/threonine protein kinase that is critically involved in the activation of NF-κB induced by TNFα [28]. Moreover, TAK1 can be activated in response to TNFα stimulation [28]. Plenty of studies showed TAK1 stabilized the endothelial function through mediating eNOS pathway [29]. Our data further confirmed the results of previous studies. Furthermore, we demonstrated that TAK1 activity could be inhibited by A20, and eNOS expression was downregulated by time-dependent TNFα stimulation, which were attenuated by TAK1 inhibition. Thus, A20 might enhance eNOS expression via promoting eNOS expression and counteract TNFα-induced eNOS uncoupling.

Endothelial NO release was blocked by TNFα treatment, but effectively rescued after AdA20 pre-treatment. Further, TAK1 inhibition by overexpression of A20 further promoted NO synthesis, suggested a crucial effect of TAK1 on A20-dependent eNOS activity and NO release. In addition, we also observed that A20 activated TAK1/P38 MAPK pathway and promoted HUVECs survival, which might be involved with promoting eNOS expression and eNOS phosphorylation. Previous investigations showed the effect of eNOS coupling on TAK1-mediated p38 MAPK pathway [29–30], the underlying mechanism for A20 in TAK1-dependent eNOS synthesis and TAK1/p38 MAPK signalling would need further investigation.

In the study we revealed A20-mediated the p38 MAPK expression was regulated by TAK1 activation in HUVECs. Previous studies indicated that oxidative stress-induced P38 MAPK was involved with eNOS promotion. Inhibition of p38 MAPK enhanced eNOS expression in human coronary artery endothelial cells. Conversely, p38 MAPK stimulated by TNFα led to a rapid dephosphorylation of eNOS and a subsequent reduction in eNOS activity and expression. According to these results, we revealed that P38 MAPK could be inhibited by A20 overexpression in which A20-mediated TAK1 activation was critically involved. Taken together, our data suggested TAK1/p38 MAPK/eNOS pathway is involved with protecting endothelial cells against atherosclerosis through depressing cellular oxidative stress stimulated by TNFα.

In summary, the study revealed that A20 might protect HUVECs against TNFα-induced cell apoptosis, enhance NO production through inhibiting TAK1/P38 MAPK. A20-mediated TAK1 inactivation abrogated P38 MAPK phosphorylation, which contributed to survival and preserved function of HUVECs. The elucidation of A20/TAK1/eNOS signalling would help to investigate the therapeutic targets for the treatment of endothelial injury and atherosclerosis.

## MATERIALS AND METHODS

### Recombinant adenoviral vectors and cultured HUVECs

To overexpress A20 we used replication-defective adenoviral vectors encompassing the full coding region of the A20 gene under the control of the cytomegalovirus promoter. A similar adenoviral vector encoding the GFP gene was used as a control. To knock down A20, TAK1 or p38 MAPK expression, three human shA20, p38 MAPK or TAK1 constructs were obtained. Next, we generated three AdshA20, p38 MAPK and TAK1 adenoviruses and selected the one that produced a significant abrogation in their expression for the next experiments using Invitrogen^R^ Block-IN system. AdsiRNA was the non-targeting control. We infected cultured HUVECs with AdA20, Adgreen fluorescent protein (AdGFP), AdsiA20, AdsiTAK1, Adsip38 MAPK or AdsiRNA, which resulted in transgenic expression without toxicity in 80-100% of the cells. For cell infection, HUVECs were cultured at a density of 1 X 10^6^ cells/well in 6-well plates and exposed to 1 X 10^8^ pfu each of virus in 1 ml of serum-free medium for 24 h. The cells were then washed and incubated in serum-containing medium for 24 h. Additional treatments are described in the figure legends.

### Cell infections

For the cell infections, 1×10^6^/well HUVEC were cultured in 6-well plates and exposed to 2×10^8^ pfu of each virus in 1 ml of serum-free medium for 24 hours. The cells were then rinsed and cultured in serum-containing media for 24 hours. The adenovirus included AdA20, AdsiA20, AdsiTAK1, Adsip38 MAPK or AdsiRNA. TNFα (20 ng/ml) was supplement to the culture of HUVEC for 2 hours.

### RNA isolation, reverse transcriptase and real-time polymerase chain reaction

Total RNA was isolated from cultured HUVECs by TRIzol (Invitrogen, USA). 500 ng RNA from per sample was reverse-transcribed for Real-time PCR assay. The primer information was shown in Table 1, Real-time PCR were performed using on a Bio-Rad IQ5 multicolor detection system by using 2 μg of cDNA. The reaction program was set as: denaturation (94 °C) for 30 s, annealing (57°C) for 30 s and extension (72°C) for 30 s. Extended at 72°C, 5 min and repeated 38 cycles. A comparative CT way was employed to measure relative quantification of RNA level. The experiments were repeated at least in triplicate.

**Table 1:** Primer information using for real-time polymerase chain reaction

Gene	Forward primer (5’-3’)	Reverse primer (5’-3’)
*eNOS*	AGCGGCTCCCAGGCCCACGA	CAGACCTGCAGTCCCGGGCA
*P38 MAPK*	CCTCTCGTAC ATCGGCGAGG	TTGATTCCAATGATGTTCTC
*GAPDH*	CCACTCTTCCACCTTCGATG	TCCACCACCCTGTTGCTGTA

Primer sequences. *eNOS*, endothelial nitric oxide synthase; *p38 MAPK*, p38 mitogen-activated protein kinase; *GAPDH*, glyceraldehyde-3- phosphate dehydrogease

### Protein extraction and western blot

All protocol was carried on as our previously described [28, 31]. Briefly, cells were lysed in RIPA lysis buffer. 20μg of protein was, respectively, soluted in SDS–polyacrylamide gel and transferred to a polyvinylidene fluoride membrane and probed with various antibodies. The membrane blots were first blocked by 5% bovine serum albumin for 30 mins, and then incubated with the primary antibody at 4°C overnight. After then, the membrane was incubated with AP-conjugated secondary antibody for 1 hour. Detection of the bands was employed through enhanced chemiluminescence (Pierce). Bands were analyzed by densitometry using Quantity One software.

### Cell apoptosis analysis by TUNEL assays

HUVECs were treated with TNFα for 2 hrs, cell apoptosis was detected using the terminal dUTP nick end labeling (TUNEL) assay (*In Situ* Cell Death Detection Kit, Roche Applied Science, IN, US). TUNEL labeling was performed following the manufacturer’s instructions. Quantification of Apoptotic Index (%) was examined by calculating TUNEL positive cells from 8 random fields per plate and was expressed as the percentage to the total cells.

### Assessment of cellular NO production

HUVECs were incubated with TNFα (20 ng/ml) at different time. At the end the experiment point, cytoplasm was obtained and 200 μl for each sample was required for sequence co-incubation with 150μl agent A and 150μl agent B. The procedure is following the manufacturer’s instructions. (NO assay kit, Multispecies, Thermo Fisher Scientific). The OD value for each sample was detected by microplate reader at 550 nm with peak absorption.

### Statistical analyses

Data are expressed as Mean±SEM. Differences among groups were determined by two-way ANOVA followed by a post hoc Tukey test. Comparisons between two groups were performed using an unpaired Student’s t-test. A value of P <0.05 was considered significant.

## Abbreviations

TAK1: transforming growth factor beta-activated kinase 1
eNOS: endothelial nitric oxide (NO) synthase
HUVECs: human umbilical vein endothelial cells
TUNEL: terminal dUTP nick end labeling
p38 MAPK: p38 mitogen-activated protein kinase
GAPDH: glyceraldehyde-3- phosphate dehydrogenase

## References

[1] Gutstein WH, Perez CA (2004). Contribution of vasoconstriction to the origin of atherosclerosis: a conceptual study. Trends Cardiovasc Med.

[2] Egholm C, Khammy MM, Dalsgaard T, Mazur A, Tritsaris K, Hansen AJ, Aalkjaer C, Dissing S (2016). GLP-1 inhibits VEGFA-mediated signaling in isolated human endothelial cells and VEGFA-induced dilation of rat mesenteric arteries. Am J Physiol Heart Circ Physiol.

[3] Huveneers S, Daemen MJ, Hordijk PL (2015). Between Rho(k) and a hard place: the relation between vessel wall stiffness, endothelial contractility, and cardiovascular disease. Circ Res.

[4] Kalani A, Kamat PK, Tyagi N (2015). Diabetic stroke severity: epigenetic remodeling and neuronal, glial, and vascular dysfunction. Diabetes.

[5] Sharma A, Sellers S, Stefanovic N, Leung C, Tan SM, Huet O, Granville DJ, Cooper ME, de Haan JB, Bernatchez P (2015). Direct endothelial nitric oxide synthase activation provides atheroprotection in diabetes-accelerated atherosclerosis. Diabetes.

[6] Wang Y, Huang Y, Lam KS, Li Y, Wong WT, Ye H, Lau CW, Vanhoutte PM, Xu A (2009). Berberine prevents hyperglycemia-induced endothelial injury and enhances vasodilatation via adenosine monophosphate-activated protein kinase and endothelial nitric oxide synthase. Cardiovasc Res.

[7] Dai X, Faber JE (2010). Endothelial nitric oxide synthase deficiency causes collateral vessel rarefaction and impairs activation of a cell cycle gene network during arteriogenesis. Circ Res.

[8] Nakayama T, Sato W, Kosugi T, Zhang L, Campbell-Thompson M, Yoshimura A, Croker BP, Johnson RJ, Nakagawa T (2009). Endothelial injury due to eNOS deficiency accelerates the progression of chronic renal disease in the mouse. Am J Physiol Renal Physiol.

[9] Carbone F, Liberale L, Bonaventura A, Cea M, Montecucco F (2016). Targeting inflammation in primary cardiovascular prevention. Curr Pharm Des.

[10] Daniel S, Patel VI, Shrikhande GV, Scali ST, Ramsey HE, Csizmadia E, Benhaga N, Fisher MD, Arvelo MB, Ferran C (2006). The universal NF-kappaB inhibitor a20 protects from transplant vasculopathy by differentially affecting apoptosis in endothelial and smooth muscle cells. Transplant Proc.

[11] Shrikhande GV, Scali ST, da Silva CG, Damrauer SM, Csizmadia E, Putheti P, Matthey M, Arjoon R, Patel R, Siracuse JJ, Maccariello ER, Andersen ND, Monahan T (2010). O-glycosylation regulates ubiquitination and degradation of the anti-inflammatory protein A20 to accelerate atherosclerosis in diabetic ApoE-null mice. PLoS One.

[12] Kaczmarek E, Bakker JP, Clarke DN, Csizmadia E, Kocher O, Veves A, Tecilazich F, O'Donnell CP, Ferran C, Malhotra A (2013). Molecular biomarkers of vascular dysfunction in obstructive sleep apnea. PLoS One.

[13] Damrauer SM, Fisher MD, Wada H, Siracuse JJ, da Silva CG, Moon K, Csizmadia E, Maccariello ER, Patel VI, Studer P, Essayagh S, Aird WC, Daniel S, Ferran C (2010). A20 inhibits post-angioplasty restenosis by blocking macrophage trafficking and decreasing adventitial neovascularization. Atherosclerosis.

[14] Endale M, Park SC, Kim S, Kim SH, Yang Y, Cho JY, Rhee MH (2013). Quercetin disrupts tyrosine-phosphorylated phosphatidylinositol 3-kinase and myeloid differentiation factor-88 association, and inhibits MAPK/AP-1 and IKK/NF-kappaB-induced inflammatory mediators production in RAW 264.7 cells. Immunobiology.

[15] Meares GP, Hughes KJ, Naatz A, Papa FR, Urano F, Hansen PA, Benveniste EN, Corbett JA (2011). IRE1-dependent activation of AMPK in response to nitric oxide. Mol Cell Biol.

[16] Brouard S, Berberat PO, Tobiasch E, Seldon MP, Bach FH, Soares MP (2002). Heme oxygenase-1-derived carbon monoxide requires the activation of transcription factor NF-kappa B to protect endothelial cells from tumor necrosis factor-alpha-mediated apoptosis. J Biol Chem.

[17] Wu H, Cheng XW, Hu L, Takeshita K, Hu C, Du Q, Li X, Zhu E, Huang Z, Yisireyili M, Zhao G, Piao L, Inoue A (2016). Cathepsin S activity controls injury-related vascular repair in mice via the TLR2-mediated p38MAPK and PI3K-Akt/p-HDAC6 signaling pathway. Arterioscler Thromb Vasc Biol.

[18] Singh BN, Shankar S, Srivastava RK (2011). Green tea catechin, epigallocatechin-3-gallate (EGCG): mechanisms, perspectives and clinical applications. Biochem Pharmacol.

[19] Omori E, Inagaki M, Mishina Y, Matsumoto K, Ninomiya-Tsuji J (2012). Epithelial transforming growth factor beta-activated kinase 1 (TAK1) is activated through two independent mechanisms and regulates reactive oxygen species. Proc Natl Acad Sci U S A.

[20] Dubey M, Nagarkoti S, Awasthi D, Singh AK, Chandra T, Kumaravelu J, Barthwal MK, Dikshit M (2016). Nitric oxide-mediated apoptosis of neutrophils through caspase-8 and caspase-3-dependent mechanism. Cell Death Dis.

[21] Mao Y, Wang J, Yu F, Li Z, Li H, Guo C, Fan X (2016). Ghrelin protects against palmitic acid or lipopolysaccharide-induced hepatocyte apoptosis through inhibition of MAPKs/iNOS and restoration of Akt/eNOS pathways. Biomed Pharmacother.

[22] Zhou HF, Yan H, Hu Y, Springer LE, Yang X, Wickline SA, Pan D, Lanza GM, Pham CT (2014). Fumagillin prodrug nanotherapy suppresses macrophage inflammatory response via endothelial nitric oxide. ACS Nano.

[23] Bi X, Niu J, Ding W, Zhang M, Yang M, Gu Y (2016). Angiopoietin-1 attenuates angiotensin II-induced ER stress in glomerular endothelial cells via a Tie2 receptor/ERK1/2-p38 MAPK-dependent mechanism. Mol Cell Endocrinol.

[24] Nguyen SD, Maaninka K, Lappalainen J, Nurmi K, Metso J, Oorni K, Navab M, Fogelman AM, Jauhiainen M, Lee-Rueckert M, Kovanen PT (2016). Carboxyl-terminal cleavage of apolipoprotein A-I by human mast cell chymase impairs its anti-inflammatory properties. Arterioscler Thromb Vasc Biol.

[25] Haasdijk RA, Den Dekker WK, Cheng C, Tempel D, Szulcek R, Bos FL, Hermkens DM, Chrifi I, Brandt MM, Van Dijk C, Xu YJ, Van De Kamp EH, Blonden LA (2016). THSD1 preserves vascular integrity and protects against intraplaque haemorrhaging in ApoE-/- mice. Cardiovasc Res.

[26] Wei HJ, Li YH, Shi GY, Liu SL, Chang PC, Kuo CH, Wu HL (2011). Thrombomodulin domains attenuate atherosclerosis by inhibiting thrombin-induced endothelial cell activation. Cardiovasc Res.

[27] Heiss C, Rodriguez-Mateos A, Kelm M (2015). Central role of eNOS in the maintenance of endothelial homeostasis. Antioxid Redox Signal.

[28] Li L, Chen Y, Doan J, Murray J, Molkentin JD, Liu Q (2014). Transforming growth factor beta-activated kinase 1 signaling pathway critically regulates myocardial survival and remodeling. Circulation.

[29] Deanfield JE, Halcox JP, Rabelink TJ (2007). Endothelial function and dysfunction: testing and clinical relevance. Circulation.

[30] Erdogdu O, Eriksson L, Xu H, Sjoholm A, Zhang Q, Nystrom T (2013). Exendin-4 protects endothelial cells from lipoapoptosis by PKA, PI3K, eNOS, p38 MAPK, and JNK pathways. J Mol Endocrinol.

[31] Li L, Chen Y, Li J, Yin H, Guo X, Doan J, Molkentin JD, Liu Q (2015). TAK1 regulates myocardial response to pathological stress via NFAT, NFkappaB, and Bnip3 pathways. Sci Rep.

